# *ADORA2A rs5760423* and *CYP1A2 rs762551* Polymorphisms as Risk Factors for Parkinson’s Disease

**DOI:** 10.3390/jcm10030381

**Published:** 2021-01-20

**Authors:** Vasileios Siokas, Athina-Maria Aloizou, Zisis Tsouris, Ioannis Liampas, Panagiotis Liakos, Daniela Calina, Anca Oana Docea, Aristidis Tsatsakis, Dimitrios P. Bogdanos, Georgios M. Hadjigeorgiou, Efthimios Dardiotis

**Affiliations:** 1 Laboratory of Neurogenetics, Department of Neurology, University Hospital of Larissa, Faculty of Medicine, School of Health Sciences, University of Thessaly, 41110 Larissa, Greece; vsiokas@med.uth.gr (V.S.); aaloizou@med.uth.gr (A.-M.A.); zitsouri@med.uth.gr (Z.T.); liampasioannes@gmail.com (I.L.); hadjigeorgiou.georgios@ucy.ac.cy (G.M.H.); 2 Laboratory of Biochemistry, Faculty of Medicine, University of Thessaly, 41110 Larissa, Greece; pliakos@med.uth.gr; 3 Department of Clinical Pharmacy, University of Medicine and Pharmacy of Craiova, 200349 Craiova, Romania; calinadaniela@gmail.com; 4 Department of Toxicology, University of Medicine and Pharmacy of Craiova, 200349 Craiova, Romania; daoana00@gmail.com; 5 Laboratory of Toxicology, School of Medicine, University of Crete, 71003 Heraklion, Greece; tsatsaka@uoc.gr; 6 Department of Rheumatology and Clinical Immunology, Faculty of Medicine, School of Health Sciences, University of Thessaly, 41110 Larissa, Greece; bogdanos@uth.gr; 7 Department of Neurology, Medical School, University of Cyprus, 1678 Nicosia, Cyprus

**Keywords:** PD, caffeine, polymorphism, genetics, *ADORA2A*, *rs5760423*, *CYP1A2*, *rs762551*

## Abstract

Background: Parkinson’s disease (PD) is the second commonest neurodegenerative disease. The genetic basis of PD is indisputable. Both *ADORA2A rs5760423* and *CYP1A2 rs762551* have been linked to PD, to some extent, but the exact role of those polymorphisms in PD remains controversial. Objective: We assessed the role of *ADORA2A rs5760423* and *CYP1A2 rs762551* on PD risk. Methods: We genotyped 358 patients with PD and 358 healthy controls for *ADORA2A rs5760423* and *CYP1A2 rs762551*. We also merged and meta-analyzed our data with data from previous studies, regarding these two polymorphisms and PD. Results: No significant association with PD was revealed (*p* > 0.05), for either *ADORA2A rs5760423* or *CYP1A2 rs762551*, in any of the examined genetic model of inheritance. In addition, results from meta-analyses yield negative results. Conclusions: Based on our analyses, it appears rather unlikely that *ADORA2A rs5760423* or *CYP1A2 rs762551* is among the major risk factors for PD, at least in Greek patients with PD.

## 1. Introduction

Parkinson’s disease (PD) is a complex neurodegenerative disorder and is characterized by premature prominent loss of dopaminergic neurons in the substantia nigra pars compacta (SNpc), resulting to dopamine deficiency [1,2]. Another hallmark of the disease (from a neuropathological aspect) is the so-called “Lewy pathology”, where the aggregated misfolded α-synuclein forms inclusions within the main neuronal body, the Lewy bodies, and the processes of neurons, the Lewy neurites [3,4]. PD is the second commonest neurodegenerative disorder following Alzheimer’s disease (AD), and in fact the proportion of patients with PD is estimated to increase by at least 50% within the next 10 years [5]. PD is 1.5-fold more frequent among males than females, while its reported prevalence varies between ethnicities from 10 to 1500 cases per 100,000 human individuals [1]; its incidence ranges from 5 to >35 new PD cases per 100,000 people [6].

The symptomatology of PD includes both motor and non-motor clinical manifestations. Thus, the dopamine deficits in the basal ganglia give way to motor symptoms that can be traditionally divided into cardinal (bradykinesia, loss of postural reflexes, rest tremor and rigidity) and secondary ones (dysarthria, hypomimia, shuffling gait, freezing, etc.) [7]. Additionally, non-motor symptoms (e.g., sleep disorders, hallucinations, and cognitive impairment to name a few) can also manifest during disease progression [8]. The course of PD is outlined by worsening of the clinical (motor and non-motor) features, while treatment-related complications can also appear in the advanced stages of the disease [9].

The pathophysiological mechanisms leading to PD remain incompletely understood [10]. Various possible neuropathophysiological procedures involved in PD have been described, such as oxidative stress, mitochondrial dysfunction and neuroinflammation [11]. Their additive action ultimately results to dopaminergic neuron death in the SNpc [12]. Few risk factors (genetic and non-genetic/environmental) have been reported to either increase or decrease PD susceptibility [13]. Regarding the environmental factors, pesticide exposure, agricultural occupation, coffee consumption, tobacco smoking, infections and prior head injury are among those known to modify PD risk [13,14,15]. There is accumulating evidence that gene–environment interactions and epigenetics can trigger the initiation of molecular processes ultimately leading to PD development, and thus further modify the risk for PD [16,17,18].

The genetic architecture of PD is indisputable. Polymeropoulos et al. (1997) performed the first linkage study where an unambiguous familial segregation of the missense mutation *A53T* across the *α-synuclein (SNCA)* gene was identified, following an autosomal-dominant inheritance pattern in PD patients with adult-onset [19]. Since then, at least 23 genetic loci and 19 causative genes have been reported in the context of PD, although accompanied by great phenotypic heterogeneity (clinical manifestations, mode of inheritance, disease progression, and age at disease onset) [20]. The genetic component of PD is also evident from genetic risk factors, derived from candidate gene association studies and genome-wide association studies (GWASs), with *SNCA*, *glucosylceramidase beta (GBA)*, *microtubule-associated protein tau gene (MAPT)* and *leucine-rich repeat kinase 2 (LRRK2)* genes considered as the commonest ones [21,22,23].

Adenosine Receptor Subtype A2a (ADORA2A) is a G-protein coupled adenosine receptor encoded by the *ADORA2A* gene. It is mainly involved in pathophysiological processes including neuroinflammation, synaptic plasticity in glutamatergic synapses and neurogenesis [24,25]. ADORA2A is expressed in the striatum, and it has been found to negatively influence the activity of dopamine D2 receptors (DRD2) [26]. Previous studies have suggested that *ADORA2A* gene polymorphisms may influence physiological responses to caffeine consumption [27,28]. Despite its apparent functional effects, very few studies have provided information regarding the mechanisms by which it exerts its effects. Moreover, in the 1-methyl-4-phenyl 1,2,3,6-tetrahydropyridine (MPTP) neurotoxin model of PD, neuroprotection resulted from the blockage of ADORA2A by caffeine [29], hinting towards a possible relation between ADORA2A and PD. Cytochrome P450 1A2 (CYP1A2), apart from other drugs, is also the main metabolizer of caffeine. It metabolizes over 90% of caffeine in paraxanthine and is located in most human brain regions [30,31,32]. The *rs762551* polymorphism appears to influence CYP1A2 function, as individuals who carry the *rs762551* seem to experience decreased CYP1A2 activity [33]. Both *ADORA2A rs5760423* and *CYP1A2rs762551* have been related, to some extent, to PD risk [34,35].

In view of the former considerations, namely certain genetic factors strongly predispose to PD, *ADORA2A rs5760423* and *CYP1A2 rs762551* polymorphisms may influence the risk of PD and previous studies examining the effect of these polymorphisms to PD have produced inconsistent results, the objective of the current study was to examine the possible crude association of *ADORA2A rs5760423* and *CYP1A2 rs762551* variants with PD, by performing a case–control study and a cumulative meta-analysis by merging our data with relevant data from previous studies.

## 2. Materials and Methods

### 2.1. Participants

Three hundred fifty-eight individuals with PD (48% male, mean age ± standard deviation (SD) = 68.07 ± 9.55 years and mean age at onset ± SD = 63.01 ± 10.12 years) and 358 healthy controls (matched for age and sex) were gathered from the Neurology Department, University Hospital of Larissa, Greece (Table 1).

The diagnosis of PD cases was carried out by consultant neurologists, based on the UK Parkinson’s Disease Society Brain Bank’s clinical criteria [8]. The main features of patients’ cohort and healthy controls have been described previously [36]. All participants provided written informed consent, while the study protocol obtained the approval of the local ethics committee.

### 2.2. Molecular Genetics

Genomic DNA was isolated from peripheral blood leucocytes by applying the salting out method [37,38,39,40]. All individuals (patients with PD and controls as well) were genotyped for *ADORA2A rs5760423* and *CYP1A2 rs762551* using the TaqMan allele specific discrimination assays on an ABI PRISM 7900 Sequence Detection System. The results were analyzed with SDS software (Applied Biosystems, Foster City, CA, USA). The genotype call rate was >98.04%.

### 2.3. Statistical Analysis

The calculation of the power of the sample was carried out with the CaTS Power Calculator [41]. The Hardy–Weinberg equilibrium (HWE) and test for association (odds ratios (ORs) with the respective 95% confidence intervals (CIs)) were carried out with SNPStats software [42], using 0.05 as the threshold for statistical significance.

To increase the power of the analysis, a cumulative meta-analysis (by merging our data with relevant data from previous studies) was performed [34,35,43,44,45] (baseline characteristics of the previous studies included in our analyses can be found in Supplementary File S1). We included studies where raw genotypic data were available or where these data could be calculated. RevMan 5.3 statistical software was utilized for the statistical analyses performed. The Z test was used to calculate the OR and the 95% CI for the effect of the *ADORA2A rs5760423* and *CYP1A2 rs762551* polymorphisms on PD. The statistical heterogeneity was computed with the Q-statistic [46] (homogeneity rejected if P_Q_ < 0.1 and/or I^2^ > 75%) [47,48]. We calculated both Mantel–Haenszel (MH) (fixed effect (FE)) and random effects (RE) [49,50,51]. The effect was tested for the effect of the heterozygosity and for the dominant and the recessive models as well.

## 3. Results

Three hundred fifty-eight individuals with PD (48% male, mean age ± standard deviation (SD) = 68.07 ± 9.55 years and mean age at onset ± SD = 63.01 ± 10.12 years) and 358 controls (matched for age and sex), were genotyped for the *ADORA2A rs5760423* and *CYP1A2 rs762551* polymorphisms. The power of our sample, with a minor allele frequency of 34% in the PD cohort, was 80.9%, with a genetic risk equal to 1.58 for the dominant mode.

The genotypic call rate was equal to 98.04% and 99.01%, for *ADORA2A rs5760423* and *CYP1A2rs762551*, respectively. No deviation from the HWE was found (*p* > 0.05) in either cases or controls, for both polymorphisms. The genotypic and allelic frequencies of the genotype polymorphisms are presented in Table 2.

No significant association was found (*p* > 0.05) for any of the examined polymorphisms. For *ADORA2A rs5760423*, the OR ranged from 0.77 to 1.19 in the over-dominant and recessive model of inheritance, respectively. The OR ranged from 0.76 (over-dominant) to 1.05 (recessive) for *CYP1A2 rs762551*. The results of the statistical analyses (ORs, CIs, and *p*-values) of both *ADORA2A rs5760423* and *CYP1A2 rs762551* regarding the risk of PD can be found in Table 3.

The cumulative statistical analysis revealed no significant results (in any assumed genetic model for both MH (FE) and RE models) for possible association between PD and *ADORA2A rs5760423* (1898 PD cases and 1950 controls; OR ranging 0.91–1.05; pz > 0.15) and/or *CYP1A2 rs762551* (4205 PD cases and 6562 controls; OR ranging 1.00–1.08; pz > 0.28). The Forest plots of the analyses for *ADORA2A rs5760423* can be accessed in Supplementary File S2, while the respective one for *CYP1A2 rs762551* in Supplementary File S3.

## 4. Discussion

The primary aim of the current study was to assess the possible implications of the *ADORA2A rs5760423* and *CYP1A2 rs762551* polymorphisms to PD risk. We initially performed a case–control study and then a meta-analysis, by gathering available published data regarding the *ADORA2A rs5760423* and *CYP1A2 rs762551* polymorphisms to the risk of PD. Based on our analyses, it seems rather unlikely that these genetic variants confer susceptibility to PD.

The ties of caffeine to PD have long been explored. An increased coffee intake has been shown to exert a protective effect, especially on genetically susceptible individuals, while caffeine compounds may even be considered as add-ons to traditional PD treatment, via its interaction with levodopa and its effect on dyskinesia and gait abnormalities [52]. Interestingly, nicotine and smoking, a habit most commonly shown to aggravate several diseases, including neurodegenerative ones, has also been described as neuroprotective in regards to PD [53]. In addition, the protective effect of caffeine seems to be dependent on sex, since caffeine and estrogen antagonize towards CYP1A2, caffeine’s main metabolizer and a known estrogen metabolizing enzyme; thus, this protective effect may not be as profound in females as in males [54]. All these facts denote a strong role of gene–environment interactions in the pathogenesis of PD, and the interest towards caffeine has been growing, particularly since it may hold therapeutical significance [52]. However, since there are gene polymorphisms that influence its metabolism and activity in the central nervous system, their association with PD definitely merits more research, which was the primary aim of this study.

In 2007, Tan et al. conducted a study exploring the role *CYP1A2 rs762551* in PD, based on the following observations; (a) caffeine (and its main metabolite paraxanthine) appears to have a neuroprotective effect, and thus may protect against PD; and (b) *rs762551* may affect the inducibility of CYP1A2, and thus influence the caffeine metabolism and its neuroprotective effect [33,44,55]. However, no proof of a connection emerged through multivariate analysis for interaction effects of caffeine with CYP1A2 and PD risk [44]. Henceforth, a number of studies attempted to investigate the role of *rs762551* in PD. *CYP1A2 rs762551* was not associated with PD susceptibility, even after the inclusion of coffee consumption in the models [56]. Increased PD risk was reported with each increasing minor allele of *CYP1A2 rs762551*, while no interaction of the *rs762551* polymorphism and caffeine intake in determining PD risk was found in the study of Kim et al. (2008) [45]. However, subsequent studies yielded different results. In particular, in 2010, Palacios et al. reported a marginal association between *CYP1A2 rs762551* C allele and PD risk [34]. Moreover, Popat et al. (2011) reported a coffee–PD association mainly among slow metabolizer homozygotes for the C allele of *rs762551* [43], results that were not replicated in the study by Hill-Burns et al. (2011) [57]. More recently, Chuang et al. (2016) reported marginal associations of *ADORA2A rs5760423* and *CYP1A2 rs762551* with PD, while *ADORA2A rs5760423* was found to significantly interact with coffee consumption in incident PD [35]. In view of the former considerations, a consensus regarding the role of *ADORA2A rs5760423* and *CYP1A2 rs762551* in PD (in correlation or not to caffeine intake) was not reached.

The *ADORA2A* gene (located at Chromosome 22 (24,417,879–24,442,357)) encodes ADORA2A, a G-protein coupled adenosine receptor, which is reported to be involved in neurogenesis, neuroinflammation and synaptic plasticity in glutamatergic synapses [24,25]. It is hypothesized that ADORA2A is implicated in PD pathogenesis as it negatively influences the activity of DRD2 receptors in the striatum [26] and appears to play a role in the MPTP neurotoxin model of PD (Chen et al. 2001). Apart from the *rs5760423*, other *ADORA2A* variants have been examined for possible association with PD phenotypes. More precisely, no associations between the *rs13306115* and PD severity [55], between *rs3032740* and PD risk [43,56] and between *rs5751876* with either PD severity or PD risk [43,58] have been reported. However, a trend for association for *ADORA2A rs2298383* and *rs3761422* and dyskinesia in PD patients has been found [59]. Additionally, *rs71651683* (a 5′ variant) and *rs5996696* (in the promoter region), across the *ADORA2A* gene, have been associated with decreased PD risk [43].

The *CYP1A2* gene (Chromosome 15 (74,748,845–74,756,607)) encodes the CYP1A2 enzyme. CYP1A2 is expressed in most human brain tissue and is the main metabolizer of caffeine (metabolizing over 90% of caffeine in paraxanthine) alongside a plethora of other drugs [30,31,32,60,61]. Apart from *rs762551*, other *CYP1A2* variants have been examined for an association with PD phenotypes. More precisely, *rs35694136*, *rs2470890*, *rs2472304*, *rs138652540*, *rs3579837*, *rs45486893* and *rs7254751* were not associated with PD [35,43,56,57,58]. However, the coffee–PD association was more robust among homozygotes for *rs2470890* [43].

Our study has some strengths worth mentioning. Firstly, the PD sample is characterized by high homogeneity, as it was collected from the same geographical area and does not include ethnically or racially distinct populations. Furthermore, we increased the power of the analysis by meta-analyzing our data with those from previous relevant studies. The main limitation is the inclusion of PD patients without screening for common PD-causative genes and sporadic PD causative factors (e.g., *SNCA*, *GBA, MAPT* and *LRRK2* genes), which might mask the effect of *ADORA2A rs5760423* and *CYP1A2 rs762551*. Moreover, the addition of potential confounding factors (especially caffeine intake) in the statistical models would have granted more robustness to our findings. Finally, the possibility that some eligible studies failed to be retrieved through our manual search is not likely, but it cannot be completely ruled out.

## 5. Conclusions

Whether *ADORA2A rs5760423* and *CYP1A2 rs762551* can be considered as genetic risk factors for PD remains debatable, although our data suggest otherwise. Larger multiethnic samples should be conducted, investigating the carriage of *ADORA2A rs5760423* and *CYP1A2 rs762551* and PD risk also including multiple genetic and environmental cofounders.

## Figures and Tables

**Table 1:** Demographic and Clinical Characteristics of PD Participants.

	PD
*n*	358
Male, *n* (%)	172
Female, *n* (%)	186
Male:Female ratio	0.92
Age at time of analysis, mean ± SD (y)	68.07 ± 9.55
Age at onset, mean ± SD (y)	63.01 ± 10.12

PD, Parkinson’s disease; SD, standard deviation.

**Table 2:** Allelic and genotype frequencies for *ADORA2A rs5760423* and *CYP1A2 rs762551* in healthy controls, in PD cases and whole sample.

Variant	Genotypes/ Alleles	Healthy Controls *n* = 358	PD *n* = 358	Whole Sample *n* = 716
*ADORA2A rs5760423*		*n* (%)	*n* (%)	*n* (%)
Genotype	G/G	108 (0.31)	121 (0.35)	229 (0.33)
	G/T	182 (0.52)	158 (0.45)	340 (0.48)
	T/T	62 (0.18)	71 (0.20)	133 (0.19)
	Missing	6	8	14
Allele	G	398 (0.57)	400 (0.57)	798 (0.57)
	T	306 (0.43)	300 (0.43)	606 (0.43)
*CYP1A2 rs762551*				
Genotype	A/A	144 (0.41)	150 (0.42)	294 (0.41)
	A/C	163 (0.46)	168 (0.47)	331 (0.47)
	C/C	47 (0.13)	37 (0.10)	84 (0.12)
	Missing	4	3	7
Allele	A	451 (0.64)	468 (0.66)	919 (0.65)
	C	257 (0.36)	242 (0.34)	499 (0.35)

PD, Parkinson’s diseases; *ADORA2A, Adenosine Receptor Subtype A2a*; *CYP1A2*, *Cytochrome P450 1A2*.

**Table 3:** Single locus analysis for association among *ADORA2A rs5760423*, *CYP1A2 rs762551* and PD in co-dominant, dominant, recessive over-dominant and log-additive modes.

Variant/Mode	Genotype	OR (95 %CI)	*p*-Value
*ADORA2A rs5760423*			
Codominant	G/G	1.00	0.22
	T/G	0.77 (0.55–1.08)	
	T/T	1.02 (0.67–1.57)	
Dominant	G/G	1.00	0.27
	T/G-T/T	0.84 (0.61–1.15)	
Recessive	G/G-T/G	1.00	0.37
	T/T	1.19 (0.82–1.74)	
Over-dominant	G/G-T/T	1.00	0.082
	T/G	0.77 (0.57–1.03)	
Log-additive	–	0.98 (0.79–1.20)	0.82
*CYP1A2 rs762551*			
Codominant	A/A	1.00	0.5
	C/A	0.99 (0.72–1.35)	
	C/C	0.76 (0.46–1.23)	
Dominant	A/A	1.00	0.67
	C/A-C/C	0.94 (0.70–1.26)	
Recessive	A/A-C/A	1.00	0.24
	C/C	0.76 (0.48–1.20)	
Over-dominant	A/A-C/C	1.00	0.73
	C/A	1.05 (0.78–1.41)	
Log-additive	–	0.91 (0.73–1.13)	0.38

PD, Parkinson’s disease; CI, confidence interval; OR, odds ratio; *ADORA2A*, *Adenosine Receptor Subtype A2a*; *CYP1A2*, *Cytochrome P450 1A2*.

## Data Availability

The data presented in this study are available on request from the corresponding author.

## Supplementary Materials

The following are available online at https://www.mdpi.com/2077-0383/10/3/381/s1, File S1: Baseline characteristics of the studies included in the meta-analysis, File S2: Forest Plots presenting the results from overall meta-analysis for theADORA2A rs5760423 and PD, File S3: Forest Plots presenting the results from overall meta-analysis for the CYP1A2 rs762551 and PD.
