# Supplementary material for: ADORA2A rs5760423 and CYP1A2 rs762551 Polymorphisms as Risk Factors for Parkinson’s Disease
Source: J Clin Med. 2021 Jan 20;10(3):381. doi: 10.3390/jcm10030381 (PMC7864159; doi:10.3390/jcm10030381)
Supplement: Supplementary file 1 [file jcm-10-00381-s001.zip › SUPPLE/Supplementary_File_S1_17.01.2021.pdf]

Table S1. Baseline characteristics of studies investigating the association of the *ADORA2A rs5760423* and *CYP1A2 rs762551* polymorphisms in PD patients and controls.

					**Cases**			**Controls**			
**Author (Year)**	**Population**	**ADORA2A rs5760423/CYP1A2 rs762551**	**Including patients positive family history/HWE test**	**Diagnosis Assessment**	**Mean age±SD/ Age of onset±SD**	**n**	**Sex Female/ male**	**Mean Age±SD**	**n**	**Sex Female/ male**	**Main Results & Comments**
Tan et al., (2007) [1]	-	no/yes	-/cases and controls	UK Brain Bank criteria	69.5 ± 10.3/63.5 ± 11.0 years	418	175/243	63.6 ± 11.3 years	468	241/227	Negative
Palacios et al., (2010) [2]	>90% Caucasian	no/yes	-/yes	Hughes AJ, Daniel SE, Lees AJ. Improved accuracy of clinical diagnosis of Lewy body Parkinson's disease. Neurology 2001;57(8):1497e9	48 (female), 60 (male)/-	298	159/139	48 (female), 60 (male)	1294	724/561	Positive
Popat et al., (2011) [3]	mixed	no/yes	yes/controls	CAPIT/Hughes criteria<br>Neurology clinic record review, neurologic exam and diagnosis confirmed by study neurologist	64.9 years/-	1325	61.1 % male	68.4 years	1735	65.4% male	Negative
Chuang et al., (2016) [4]	Non-Hispanic whites Danish	yes /yes	yes/controls	UK Brain Bank criteria (Hughes AJ, Daniel SE, Kilford L, et al. Accuracy of clinical diagnosis of idiopathic Parkinson's disease: a clinicopathological study of 100 cases. J Neurol Neurosurg Psychiatry. 1992;55(3):181–184.)<br><br>Gelb DJ, Oliver E, Gilman S. Diagnostic criteria for Parkinson disease. Arch Neurol. 1999;56(1):33–39	61.1±9.5/-	1556	59.9 % male	61.3±9.7 years	1606	60.7% male	Marginal Associations
Kim et al., (2018) [5]	Derived from 2 large prospective cohorts (NHS, HPFS)	no/yes	-/yes	Based on medical records were requested from PD cases (after 2003 records reviewed by a neurologist specializing in movement disorders)	-	-	-	-	-	-	CYP1A2 rs762551 was associated with an increased risk in the NHS, but not in the HPFS

PD, Parkinson's disease; ADORA2A, Adenosine Receptor Subtype A2a; CYP1A2, Cytochrome P450 1A2; NHS, Nurses' Health Study; HPFS, the Health Professionals Follow-up Study; OR, odds ratio; CI, confidence interval.

REFERENCES

1. Tan EK, Chua E, Fook-Chong SM, Teo YY, Yuen Y, Tan L, et al. Association between caffeine intake and risk of Parkinson's disease among fast and slow metabolizers. Pharmacogenetics and genomics. 2007;17(11):1001-5.
2. Palacios N, Weisskopf M, Simon K, Gao X, Schwarzschild M, Ascherio A. Polymorphisms of caffeine metabolism and estrogen receptor genes and risk of Parkinson's disease in men and women. Parkinsonism & related disorders. 2010;16(6):370-5.
3. Popat RA, Van Den Eeden SK, Tanner CM, Kamel F, Umbach DM, Marder K, et al. Coffee, ADORA2A, and CYP1A2: the caffeine connection in Parkinson's disease. European journal of neurology. 2011;18(5):756-65.
4. Chuang YH, Lill CM, Lee PC, Hansen J, Lassen CF, Bertram L, et al. Gene-Environment Interaction in Parkinson's Disease: Coffee, ADORA2A, and CYP1A2. Neuroepidemiology. 2016;47(3-4):192-200.
5. Kim IY, O'Reilly É J, Hughes KC, Gao X, Schwarzschild MA, McCullough ML, et al. Interaction between caffeine and polymorphisms of glutamate ionotropic receptor NMDA type subunit 2A (GRIN2A) and cytochrome P450 1A2 (CYP1A2) on Parkinson's disease risk. Movement disorders : official journal of the Movement Disorder Society. 2018;33(3):414-20.
